# Evidence-based systematic review of removal of peripheral arterial catheter in critically ill adult patients

**DOI:** 10.1186/s12871-024-02458-0

**Published:** 2024-02-26

**Authors:** Hongju Wang, Lihuan He, Chun Han, Jianhong Wan

**Affiliations:** 1 Department of Thoracic Surgery, The Second Hospital of Shandong University, Jinan, 250033 China; 2 Department of Cardiovascular Surgery, The Second Hospital of Shandong University, 247 Beiyuan Street, Jinan, Shangdong 250033 China

**Keywords:** Evidence-based nursing, Evidence summary, Removal, Peripheral arterial manometry catheter

## Abstract

**Objective:**

To evaluate and summarize literature pertaining to evidence of peripheral arterial catheterization in adults, and to provide a reference for clinical practice.

**Methods:**

We undertook a systematic review of literature on the removal of peripheral arterial manometric catheters in adult patients from various sources such as UpToDate, BMJ, National Institute for Health and Care Excellence (NICE), Medlive, Cochrane Library, Joanna Briggs Institute (JBI) Evidence-based Health Care Center Database, CINAHL, PubMed, Wanfang Data, VIP, and other databases. The retrieval time was set as from the establishment of the database till August 30, 2021. We screened the studies that fulfilled the inclusion criteria, evaluated their quality, and retrieved and summarized such articles.

**Results:**

The review included 8 articles: 1 clinical decision, 3 guidelines, 2 evidence summaries, 1 systematic review, and 1 expert consensus. In all, 17 pieces of strong evidence were collected and extracted based on the following 5 dimensions: assessment of removal timing, preparation before removal, removal procedure, compression time, and key points after removal.

**Conclusions:**

The removal of a peripheral arterial manometry catheter requires careful consideration by medical professionals. In order to increase the removal standardization rate and decrease the incidence of clinical complications, standardized procedures and training need to be developed.

**Supplementary Information:**

The online version contains supplementary material available at 10.1186/s12871-024-02458-0.

## Background

Percutaneous arterial puncture and insertion of an indwelling catheter into the artery lumen is known as arterial catheterization (AC). Its widespread clinical application can be attributed to its utility as a convenient channel for intermittent blood sampling in laboratory testing and its prevalence in the monitoring of invasive arterial blood pressure. [1, 2] Patients in critical care often have indwelling catheters placed to measure their blood pressure continuously. Continuous arterial catheter-based monitoring was found to be more effective at detecting hypotension than oscillometric-based monitoring with a blood pressure cuff in a study of patients undergoing cardiac surgery [3]. The evaluation of respirophasic variations in the arterial pressure waveform to predict fluid responsiveness is made possible by continuous monitoring of arterial blood pressure, which also allows for frequent blood sampling and the detection of abnormal arterial waveform patterns [4]. All of these elements work together to make nursing and other healthcare tasks easier and more effective [5]. About 8 million arterial catheters are used annually in the United States, while in Europe the number is closer to 2.5 million [6]. The technique for insertion of an indwelling arterial catheter is quite mature [7]. Numerous studies have examined arterial catheterization, most of which have compared different insertion techniques. However, arterial catheter removal is discussed in only a minority of articles [8], and the process is complex. The current procedural standards for performing peripheral arterial catheter removal in clinical practice are deficient, and there are complications such as hemorrhage and hematoma due to improper removal of the arterial catheter [9]. Local hematoma occurred during catheter insertion in 4.5% of patients and during removal in 1.2% of patients, according to a previous study [10]. Also, local ischemia was observed in 0.2% of the patients during catheter removal, characterized by symptoms such as pain, localized discoloration, abnormal sensations, swelling, and coolness in both the area near the arterial catheter insertion site and the distal region of the catheter insertion site [11, 12]. The purpose of this study was to compile the current state of knowledge on the safe removal of peripheral arterial catheters in adults in order to inform clinical nursing practice and increase patient safety.

## Materials and methods

### Literature search strategy

Our research questions for this literature review followed the PICO format. Information about the PICO format and the indexing terms can be found in the Supplementary Tables 1 and Supplementary Material 1, respectively. Between January 1, 2000, and August 30, 2021, a number of electronic databases were used.

A flowchart of the process of selection has been shown in Supplementary Fig. 1.

### Inclusion and exclusion criteria

Inclusion criteria: Studies whose participants were adult patients with indwelling peripheral arterial catheters, aged ≥ 18 years. The study must involve peripheral arterial catheter removal.

The types of studies included guidelines, evidence summary, clinical decision, systematic review, meta-analysis, and expert consensus. The language was restricted to Chinese and English. Exclusion criteria: (1) literature was in the form of proposals or updated literature; (2) literature with unrelated topic; (3) literature with study population failed to meet the inclusion criteria; (4) literature without peripheral arterial catheter-related content; (5) literature with results not meeting the criteria.

### Process of evaluating the quality of literature

We set up an evidence team, and three researchers trained in evidence-based nursing independently evaluated the literature to be included. When there were differences of opinion, the team discussed and arrived at the final decisions. Conflicting conclusions with respect to evidence from different sources were resolved using the principle of prioritizing evidence-based, high-quality evidence, and newly published literature from subject experts.

### Criteria for evaluating the quality of literature

We used the Appraisal of Guidelines for Research and Evaluation Instrument 2012 (AGREEII) to evaluate the quality of the guidelines [13]. This scale consists of 6 quality domains, 23 key items, and 2 global rating items. The score for each item ranges from 1 to 7 points, with 1 = strongly disagree and 7 = strongly agree. The score for each domain was normalized to the percentage of the highest possible score in that area and calculated as follows: [(actual score – minimum possible score)/(maximum possible score – minimum possible score)] *100%. Based on the scores of each domain of the guidelines and the final judgment of researchers, the recommendations of the guidelines were categorized into three levels. Grade A recommendation refers to direct recommendation with no need to revise, with scores ≥ 60% in all six domains. Grade B recommendation refers to recommendation with modification and improvement, with a score of < 60% and a score of ≥ 30% obtained in more than 3 domains. Grade C recommendation refers to no recommendation, with a score of ≤ 30% obtained in more than 3 domains.

The quality of clinical decisions was evaluated by tracing the original literature where the evidence was located and evaluating it based on the type of literature. The evidence summary was evaluated using the CASE worksheet, which consists of 10 items with response categories of “Yes,” “Partially yes,” and “No.” The quality of the systematic review was assessed using the systematic review evaluation tool (AMSTAR 2) [14], which consists of 16 items (Supplementary Table 2). The evaluation conclusion was “Yes” when it fully met the evaluation criteria, “Partially yes” when it partly met the standard, and “No” when no relevant information was reported. The expert consensus was assessed using the JBI expert consensus evaluation tool (2015 edition) [15], and the 6 domains were evaluated as “Yes,” “No,” “Unclear,” and “Unsuitable” (Supplementary Table 3).

### Process of evaluating the quality of evidence

Members of the evidence team evaluated the evidence in the included literature for feasibility, appropriateness, clinical significance, and validity using the FAME criteria. The evidence was graded using the JBI evidence pre-classification and evidence recommendation grading system (2014 edition) (Supplementary Table 4) [16]. The evidence was compiled and classified according to the theme. In case of any ambiguity, the evidence team discussed and agreed on the final decision.

## Results

### General description of the included literature

In all, 623 articles related to secondary resources were initially retrieved, and 8 articles were finally identified after excluding duplicates, interpretations, and those with inconsistent themes. The final review consisted of 1 clinical decision [4], 3 guidelines [17–19], 2 evidence summaries [20, 21], 1 systematic review [22], and 1 expert consensus [23] (Table 1). A NICE guideline [19], a Medlive guideline [18], and a PubMed database guideline [17] were used in this study. It was determined using the AGREE II that all three pieces of literature and source materials were of sufficient quality to be included in the final evaluation. Overall, the quality of the evidence that was summarized for the quality assessment was high [21], hence it was included (Table 2). Inclusion was determined to be warranted after the AMSTAR 2 assessment of the systematic review revealed its high quality [22]. Using the JBI expert consensus assessment tool (2015), we found that the literature was of high quality and included it in the study [23]. 

**Table 1:** Overview of articles included in the review

Serial number	Article	Article source	Nature of the article	Research topic	Date of publication
1	Gilles Clermont et al. [4]	UpToDate	Clinical decision	Arterial catheterization in invasive blood pressure monitoring: extubation	2020
2	Gorski LA et al. [15]	PUBMED	Guidelines	Vascular access device removal	2021
3	O’Grady NP et al. [16]	Medlive	Guidelines	Practical suggestion of catheter	2011
4	Loveday HP et al. [17]	NICE	Guidelines	Catheter replacement strategy	2014
5	Wang Yi et al. [18]	Wangfang data	Evidence summary	Timing of peripheral arterial catheter extubation	2020
6	Guo Han Painting et al. [19]	Wangfang data	Evidence summary	Timing of peripheral arterial catheter extubation	2021
7	O’Horo JC et al. [20]	PUBMED	System review	Evaluation of arterial catheter extubation	2014
8	Ma Hong et al. [21]	Chinese anesthesia	Expert consensus	Complications and treatment of pressure monitoring of radial artery puncture catheterization	2017

**Table 2:** Results of the AGREE II evaluation of guidelines included in the review

Guidelines	Standardized scores in each domain						Number of domains with standardized score > 60% (number)	Number of domains with standardized score < 30% (number)	Recommended level
	Scope and purpose	People involved	Strictness	Clarity	Applicability	Independence			
Gorski LA et al. [14]	97.22	94.44	97.92	94.44	56.25	91.67	6	0	A
O’Grady NP et al. [15]	100	86.11	45.83	94.44	60.42	70.83	5	0	B
Loveday HP et al. [16]	100	91.66	98.96	97.22	54.17	100	5	0	B

### Description of evidence and summary

The evidence summary was compiled by collecting data from the eight articles that were included in the review. For this study, we used the Australian JBI Evidence-based Health Care Center Evidence Pre-grading System (2014) to determine the grade of the evidence we included. According to the type of research design, the grade of evidence was divided into grade 1–5, from high to low. The evidence team determined the recommendation level of evidence and made a recommendation of grade A and grade B based on the validity, feasibility, appropriateness, and clinical significance of JBI’s FAME evidence criteria (Table 3).

**Table 3:** Results of CASE worksheet evaluation for evidence summaries included in the review

Evaluation item	Wang Yi et al [17]	Guo Han Hua et al [18]
1. Is the scope and application object of evidence summary specific?	Yes	Yes
2. Is the author of the evidence summary clear and transparent?	Yes	Yes
3. Is the reviewer or editor of the evidence summary clear and transparent?	Yes	Yes
4. Is the retrieval method transparent and comprehensive?	Yes	Yes
5. Is evidence graded and the grading system used transparent?	Yes	Yes
6. Are the recommendations clear?	Yes	Yes
7. Is the citation of the recommended opinion appropriate?	Partially yes	Yes
8. Are recommendations time-sensitive?	No	No
9. Does the evidence summary avoid potential bias?	Partially yes	Partially yes
10. Does this summary of evidence apply to your patient?	Yes	Yes
Additional entry: Was the quality of the included literature evaluated?	Yes	Yes

### Evidence summary

By combining and classifying the evidence, we arrived at the summary of the final best evidence for removal of peripheral arterial catheter. The content included 17 pieces of strong evidence based on the following 5 dimensions: assessment of removal timing, preparation before removal, removal procedure, compression time, and key points after removal (Table 4).

**Table 4:** Summary of evidence for peripheral arterial manometry catheter removal in adult patients

Evidence Topic	No.	Evidence description	Level of evidence	Recommended level
Assessment of removal timing	1	The necessity of indwelling artery catheter is evaluated daily to fully assess the benefits and risks and should be removed as soon as unnecessary [13–15, 17–19].	4c	A
	2	There are obvious signs of infection, the catheter is not working effectively, and the arterial catheter should be removed immediately when complications occur (blockage, hematoma, circulatory disturbance) or when it is no longer needed [14, 16–18, 20].	4c	A
	3	If the aseptic technique for the insertion of an arterial catheter in an emergency is not strictly followed, the catheter should not be indwelled for more than 48 h [15, 17].	3e	B
Preparation before removal	4	Check the international normalized ratio, partial thromboplastin time, and platelet count before peripheral arterial catheter removal and confirm the use of drugs that interfere with coagulation and platelet function of the patients [13].	5b	B
	5	When the platelet count is < 50 × 10^9^/L, the activated partial thromboplastin time is > 1.3 times the normal value, and/or the international normalized ratio is > 1.8, correction using blood products are recommended to use [24].	5b	B
	6	Wear sterile gloves and PPE after washing hands before arterial catheter removal [13].	5b	B
	7	Clean the catheter site with chlorhexidine after removal of the dressing [13, 14, 19].	1b	A
	8	Flush artery catheter before removal. Pay attention to avoid blood spatter during removal [13].	5c	B
Removal procedure	9	Place a sterile dressing at the site of arterial puncture and compress both the artery and skin puncture sites. Slowly pull out the catheter [13, 14].	4b	B
	10	Simultaneously press the arterial puncture point and skin puncture point during removal to achieve hemostasis by manual compression [13, 14].	5c	B
Compression time	11	Generally, the radial artery should be continuously pressed for 5 min after removal, and the femoral artery should be pressed for 10 min after removal [13].	5b	B
	12	If there is blood seepage after the compression time, check again after compressing for 5 min [13].	5b	B
	13	If the international normalized ratio, partial thromboplastin time, and platelet count are abnormal, or the patient received antiplatelet therapy, the compression time should be extended by 50–100% [13].	5b	B
Points after removal	14	Check the catheter after removal to ensure integrity. Compress the distal end of the catheter if the catheter breaks [13, 14, 16].	5c	B
	15	In patients with femoral artery puncture catheterization, the hip should not be moved for 2 h after catheter removal [13, 20].	5c	B
	16	Patients were told to avoid holding the puncture-side limb forcefully or performing non-invasive blood pressure measurement within 2 h after removal. Closely observe whether local blood seepage and subcutaneous hematoma were formed [13, 20].	5b	B
	17	Assess and record the pulse at the puncture site and its distal end after removal. Notify the physician if there is any abnormality [13, 14, 16].	4c	B

## Discussion

### Preparation for the removal of peripheral arterial catheter

Peripheral arterial catheters are widely used in intensive care units [10]. However, related research is relatively limited compared to that of deep vein catheters. The healthcare community needs to recognize and fill this knowledge gap. Despite its usefulness, peripheral arterial catheterization is not without risk. As a result, extreme care must be taken throughout, beginning with the insertion and ending with the removal. Clinicians can improve efficiency and the quality of care for their patients by adopting a standardized, evidence-based approach [25]. The evidence firstly emphasizes the assessment of the indications for removal. Current evidence suggests that routine replacement of the peripheral arterial catheter is not recommended for the prevention of catheter-related infections [14], and the arterial catheter is only removed or replaced when evidence 1–3 is presented. Peripheral arterial catheters should be removed as soon as the risk of indwelling is assessed to outweigh the benefit [24]. In addition, catheters should be removed when their continued use is not essential to patient care [4], as longer catheter dwelling time is associated with an increased risk of infection [18]. 

When the platelet count is < 50 × 10^9^/L, the activated partial thromboplastin time is > 1.3 times the normal value, and/or the international normalized ratio is > 1.8, correction using blood products are recommended to use [26]. 

Irrespective of where the arterial catheter is located, standard precautions such as the use of PPE, wearing sterile gloves, and washing hands must be taken [4]. Based on the JBI FAME principles, evidence 5 was revised to “Perform arterial catheter removal for patients with coagulation disorders, thrombocytopenia, or platelet dysfunction in the presence of a physician” and evidence 6 was revised to “Wear sterile gloves and PPE after washing hands before arterial catheter removal.”

### Standard operating procedure for peripheral arterial catheter removal

Aseptic techniques must be used during removal of peripheral arterial catheters, and care must be taken to avoid splashing blood. The arterial catheter should be flushed prior to removal, and prepared for safe removal. Evidence 10 “Simultaneously press the arterial puncture point and skin puncture point during removal to achieve hemostasis by manual compression.” Even with direct arterial puncture, there is a distance between the skin puncture site and the arterial puncture site [4]. To remove the catheter, it is recommended to disinfect the skin puncture site of the catheter with chlorhexidine and apply a sterile dressing; pressure should be applied over both the arterial puncture and the skin puncture sites, and the catheter slowly withdrawn while maintaining pressure [4]. 

### Post-removal of peripheral arterial catheters

Post-removal precautions are often ignored [26], and Evidence 15–17 provide a detailed description of the concerns after removal of the peripheral arterial catheter. Medical staff need to place greater impetus on the systematic removal of peripheral arterial catheters. In case of a radial arterial catheter, pressure must be applied for 5 min after removal, and for at least 10 min in case of a femoral arterial catheter. If blood flow persists after the designated pressure time, apply pressure for an additional 5 min [4]. A previous study [27] has indicated that both manual compression and traditional radial artery hemostasis device can effectively achieve hemostasis. The use of a patented hemostatic device during the removal of peripheral catheters via the radial artery not only reduces the incidence of radial artery occlusion but also significantly decreases the workload for nursing staff.

However, most evidence comes from recommendations for coronary intervention through the distal radial artery route [28], rather than recommendations from radial artery cannulation for arterial pressure measurement. More evidence will be needed from the use of hemostasis devices during arterial catheter removal in invasive blood pressure monitoring.

Avoid repetitive examination of the puncture site to prevent prolonged bleeding. Evidence 14: Inspect the site to ensure the catheter is intact after catheter removal. If the catheter is broken, pressure should be applied to the proximal end of the catheter insertion site [4, 17, 19]. In the case of catheter fragmentation, pressure should be applied above the entry point on the skin. Catheter fragment embolization may obstruct distal limb circulation. After femoral arterial catheter removal, the limb should not be moved for up to two hours. Fifteen minutes after catheter removal, the puncture site and distal pulses should be rechecked to confirm the absence of local hematoma or signs of limb ischemia [23]. 

### Raising awareness of peripheral arterial catheter insertion and removal among healthcare professionals

A survey conducted in China in 2022 showed that 40.0–45.0% of ICU nurses lacked the relevant knowledge about the use and evaluation of arterial catheters [29]. In other words, there is considerable scope for improving the cognitive ability of ICU nurses with respect to maintenance and indwelling peripheral arterial catheter removal. Peripheral arterial catheterization and removal are invasive procedures, and an improperly conducted procedure may cause complications such as bleeding, thrombosis, catheter-related bloodstream infection, and distal limb ischemia [30]. Effective arterial catheterization intervention is a crucial component in the nursing of critically ill patients [31]. Due to lack of awareness, peripheral arterial catheterization and removal have not received due attention. In this study, we summarized the evidence for peripheral arterial catheter removal, in order to effectively prevent complications during invasive arterial pressure monitoring and improve patient monitoring quality.

## Conclusion

Comprehensive practical evidence on the assessment of arterial catheter removal, pre-removal preparations, removal procedures, and post-removal considerations can facilitate clinical nurses in implementing evidence-based practices and improving their clinical abilities [32]. In this study, we summarized the evidence pertaining to peripheral arterial catheter removal in critically ill adult patients and provided evidence-based practices for intensive care staff. Our findings suggest that in order to effectively ensure patient safety, health care professionals must carefully evaluate the appropriateness and feasibility of each piece of evidence, taking into account factors such as the department environment and patient willingness, and then apply the evidence in clinical practice.

### Electronic supplementary material

Below is the link to the electronic supplementary material.

Supplementary Material 1

Supplementary Material 2

Supplementary Material 3

Supplementary Material 4

Supplementary Material 5

Supplementary Material 6

Supplementary Material 7

## Data Availability

The datasets used and/or analysed during the current study available from the corresponding author on reasonable request.
