# Supplementary figures and images for: Evidence-based systematic review of removal of peripheral arterial catheter in critically ill adult patients
Source: BMC Anesthesiol. 2024 Feb 26;24:79. doi: 10.1186/s12871-024-02458-0 (PMC10895724; doi:10.1186/s12871-024-02458-0)

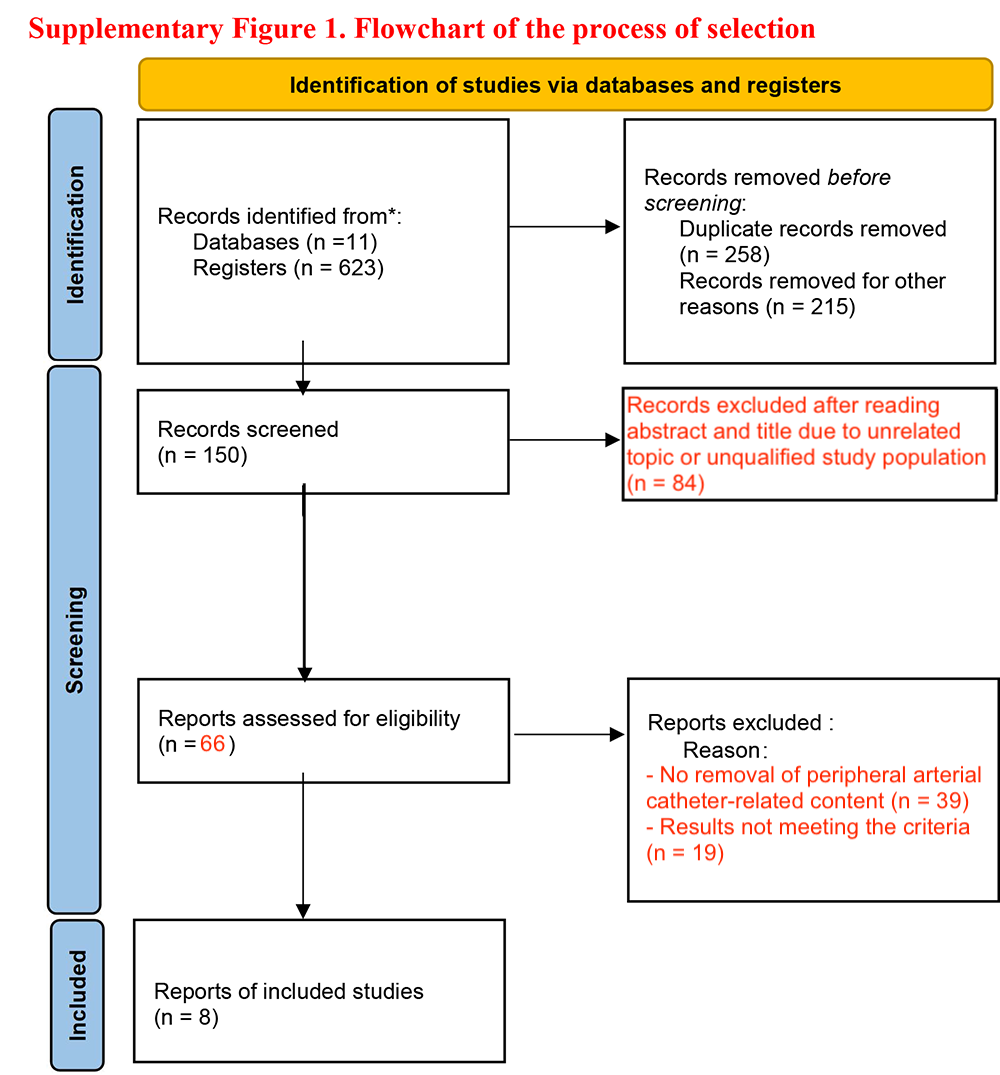

Supplement: Supplementary file 2 — Supplementary Material 2 [file 12871_2024_2458_MOESM2_ESM.png]
