# Supplementary material for: Evidence-based systematic review of removal of peripheral arterial catheter in critically ill adult patients
Source: BMC Anesthesiol. 2024 Feb 26;24:79. doi: 10.1186/s12871-024-02458-0 (PMC10895724; doi:10.1186/s12871-024-02458-0)
Supplement: Supplementary file 3 — Supplementary Material 3 [file 12871_2024_2458_MOESM3_ESM.docx]

**Supplementary Material 1. The searching strings**

We retrieved articles based on the "6S" evidence model. The databases we covered in our literature search were: UpToDate, BMJ, National Institute for Health and Care Excellence (NICE), Yimaitong, Cochrane Library, Joanna Briggs Institute (JBI) Evidence-based Health Care Center Database, CINAHL, PubMed, Wanfang Data, VIP, and other secondary resources on peripheral arterial catheter removal in adult patients. We used the following English search terms: "arterial catheter*/arterial line/catheter, indwelling/Catheterization, Peripheral*/arterial cannulation/vascular access devices/blood gas analysis"AND"removal"AND"meta-analysis/guideline/evidence summary/systematic review/system overview/clinical practice guidelines/recommended practice". The Chinese search terms we used were: “arterial canal/artery place pipe/Arterial pressure measuring tube/Vascular access/Vascular catheterization/blood gas analysis”AND“pull out/remove/tube drawing /extract”AND“hemorrhage/infect/embolism/risk/stop up/complication/safe/accident/adverse event ”AND“Meta analysis /guide/Summary of evidence/systems assessment/systematic review/specialist consensus/clinical decision” Subject headings and text-word searches were both used for retrieval
