# Supplementary material for: Evidence-based systematic review of removal of peripheral arterial catheter in critically ill adult patients
Source: BMC Anesthesiol. 2024 Feb 26;24:79. doi: 10.1186/s12871-024-02458-0 (PMC10895724; doi:10.1186/s12871-024-02458-0)
Supplement: Supplementary file 4 — Supplementary Material 4 [file 12871_2024_2458_MOESM4_ESM.docx]

**Supplementary Table 1. The PICO framework**

| **Population** | **Intervention** | **Control** | **Primary outcome** | **Literature type** |
| --- | --- | --- | --- | --- |
| Adult patients with retained arterial catheter | Arterial catheter removal | — | Bleeding | Guideline |
|  |  |  | Infection | Evidence summary |
|  |  |  | Embolization | Systematic evaluation |
|  |  |  | Safety events | Expert consensus |
|  |  |  |  | Mata-analysis |
|  |  |  |  | Clinical decision making |
