# Supplementary material for: Evidence-based systematic review of removal of peripheral arterial catheter in critically ill adult patients
Source: BMC Anesthesiol. 2024 Feb 26;24:79. doi: 10.1186/s12871-024-02458-0 (PMC10895724; doi:10.1186/s12871-024-02458-0)
Supplement: Supplementary file 5 — Supplementary Material 5 [file 12871_2024_2458_MOESM5_ESM.docx]

**Supplementary Table 2.** **AMSTAR 2 Items**

| Item No. | Item content | Yes | Partial Yes | No |
| --- | --- | --- | --- | --- |
| 1 | Did the research questions and inclusion criteria for the review include the components of PICO? |  |  |  |
| 2 | Did the report of the review contain an explicit statement that the review methods were established prior to the conduct of the review and did the report justify any siqnificant deviatons from the protocol? |  |  |  |
| 3 | Did the review authors explain their selection of the study designs for inclusion in the review? |  |  |  |
| 4 | Did the review authors use a comprehensive literature search strategy? |  |  |  |
| 5 | Did the review authors perform study selection in duplicate? |  |  |  |
| 6 | Did the review authors perform data extraction in duplicate? |  |  |  |
| 7 | Did the review authors provide a list of excluded studies and justify the exclusions? |  |  |  |
| 8 | Did the review authors describe the included studles in adequate detall? |  |  |  |
| 9 | Did the revlew authors use a satisfactory technique for assessing the risk of blas(RoB) in individual studies that were included in the review? |  |  |  |
| 10 | Did the revlew authors report the sources of funding for the studies included in the review? |  |  |  |
| 11 | If Meta-analysls was performed, did the review authors use appropriate method for statistica combination of results? |  |  |  |
| 12 | If Meta-analysls was performed, did the review authors assess the potential impact of RoB in individual studies on the result of Meta-analysis or other evidence synthesis? |  |  |  |
| 13 | Did the review authors account for RoB in individual studes when interpreting/discussing the result of the review? |  |  |  |
| 14 | Did the review authors provide a satisfactory explanation for, and discussion of, any heterogeneity observed in the result of the review? |  |  |  |
| 15 | If they performed quantitative synthesls, did the review authors carry out an adequate investigation of publication blas (small study blas and discuss its likely impact on the result of the review? |  |  |  |
| 16 | Did the review authors report any potential source of conflict of interest, including any funding they received for conducting the review? |  |  |  |
