# Supplementary material for: Evidence-based systematic review of removal of peripheral arterial catheter in critically ill adult patients
Source: BMC Anesthesiol. 2024 Feb 26;24:79. doi: 10.1186/s12871-024-02458-0 (PMC10895724; doi:10.1186/s12871-024-02458-0)
Supplement: Supplementary file 6 — Supplementary Material 6 [file 12871_2024_2458_MOESM6_ESM.docx]

**Supplementary Table 3. 2015 edition of JBI Expert Consensus Evaluation tool**

| Evaluation Item | Evaluation Outcome | | | |
| --- | --- | --- | --- | --- |
|  | Yes | No | Not clear | Not applicable |
| 1. Are the sources of the ideas clearly marked? |  |  |  |  |
| 2. Did the ideas come from influential experts in the field? |  |  |  |  |
| 3. Are the ideas presented centered on the interests of the relevant population? |  |  |  |  |
| 4. Are the stated conclusions based on the analysis? Are ideas expressed logically? |  |  |  |  |
| 5. Does it refer to other existing literature? |  |  |  |  |
| 6. Are there any inconsistencies between the ideas presented and the previous literature? |  |  |  |  |
