# Supplementary material for: Evidence-based systematic review of removal of peripheral arterial catheter in critically ill adult patients
Source: BMC Anesthesiol. 2024 Feb 26;24:79. doi: 10.1186/s12871-024-02458-0 (PMC10895724; doi:10.1186/s12871-024-02458-0)
Supplement: Supplementary file 7 — Supplementary Material 7 [file 12871_2024_2458_MOESM7_ESM.docx]

**Supplementary Table 4. Australian JBI Evidence-based Health Care Center Evidence Pre-grading System (2014)**

| Level of evidence | Type of evidence | Description |
| --- | --- | --- |
| Level 1 | RCT/Other experimental studies | 1a- Systematic Review of multiple RCTS |
|  |  | 1b- Systematic review of multiple RCTS and other intervention studies |
|  |  | 1c- Single RCT |
|  |  | 1d- Quasi-RCT |
| Level 2 | Experimental like study | 2a- Systematic review of multiple experimental studies |
|  |  | 2b- A systematic review of multiple experimental studies and other low-quality intervention studies |
|  |  | 2c- A single prospective quasi-experimental study with a control group 2d- before and after control, retrospective control quasi-experimental study |
| Level 3 | Observational - analytical research | 3a- Systematic Review of multiple cohort studies |
|  |  | 3b- A systematic review of multiple cohort studies and other low-quality observational studies |
|  |  | 3c- A single cohort study with a control group |
|  |  | 3d- Single case control study |
|  |  | 3e- Single observational study without control group |
| Level 4 | Observational - descriptive research | 4a- Systematic review of multiple descriptive studies |
|  |  | 4b- A single cross-sectional study |
|  |  | 4c- Case series study |
|  |  | 4d- Case studies |
| Level 5 | Expert opinions, basic research | 5a- Systematic evaluation of expert opinion |
|  |  | 5b- Expert Consensus |
|  |  | 5c- Basic research, individual expert opinion |
